# Brentuximab vedotin as monotherapy for unresectable breast implant‐associated anaplastic large cell lymphoma

**DOI:** 10.1002/ccr3.2142

**Published:** 2019-04-11

**Authors:** Anthony Stack, Isaac Levy

**Affiliations:** ^1^ Department of Internal Medicine Temple University Hospital Philadelphia Pennsylvania; ^2^ Millennium Oncology Pembroke Pines Florida

**Keywords:** ALCL, breast implant‐associated anaplastic large cell lymphoma, breast lymphoma, brentuximab vedotin, lymphoma

## Abstract

BI‐ALCL is a rare CD30+ T‐cell malignancy, which is known to complicate textured breast implants. The CD30‐targeting immunoconjugate, brentuximab vedotin, has been suggested for invasive BI‐ALCL; however, its efficacy for unresectable BI‐ALCL has not been demonstrated. We present a case of unresectable BI‐ALCL, which was successfully treated with brentuximab vedotin.

## 1 INTRODUCTION

Breast implant‐associated anaplastic large cell lymphoma (BI‐ALCL) is a rare, CD30+ T‐cell malignancy, which forms within the fibrous capsule surrounding breast implants. It typically presents as a localized intracapsular seroma, approximately one decade after implantation.^1^ The pathogenesis of BI‐ALCL is poorly understood. Reason for implantation (reconstructive vs cosmetic), type of implant fill (silicone vs saline), or implant size does not seem to influence risk; however, studies have implicated implant texturing as a possible contributor.^2^, ^3^

While surgical excision has become the standard of care for BI‐ALCL localized to the implant capsule, the optimal management of invasive disease has yet to be elucidated.^6^ Recently published consensus guidelines by the National Comprehensive Cancer Network (NCCN) have suggested the CD30‐targeting immunoconjugate, brentuximab vedotin (BV) as an alternative to cytotoxic chemotherapy for invasive BI‐ALCL; however, to our knowledge, application of BV for unresectable, locally invasive Bi‐ALCL has not been demonstrated in the literature.^7^

## 2 CASE DESCRIPTION

A 73‐year‐old woman presented with one month of progressive pain and swelling in her right breast. She had a past medical history of right breast cancer sixteen years prior, which had been treated with lumpectomy and chemoradiation in Colombia, followed by bilateral textured silicone breast implant placement.

Breast MRI showed that the right breast implant had been deformed by a complex effusion within the fibrous implant capsule, giving it the appearance of rupture on ultrasound. Extending superiorly from the right implant capsule was a mass, measuring up to 8 cm and invading both the chest wall and pleura. While some simple fluid extended across the midline to the medial aspect of the left breast implant, there was no suspicious enhancement to suggest left breast involvement.

A core needle biopsy of the mass was performed. Sections showed neoplastic infiltrate comprised of large malignant cells with round, oval and irregular nuclei, finely stippled chromatin, conspicuous nucleoli and abundant pale, vacuolated cytoplasm. Tumor cells were associated with a rich mixed inflammatory infiltrate comprised of small T and B lymphocytes, many eosinophils and histiocytes. Tumor cells were noted to infiltrate skeletal muscle and other soft tissues. Immunohistochemistry showed diffuse positivity for CD45, CD30, CD43, CD4, MUM‐1, and very weak positivity for CD2. The Ki67 proliferative index in tumor cells was high, approaching 90%. Tumor cells were negative for CD79a, PAX5, CD20, CD8, CD56, CD3, EMA, CD34, CD5, ALK‐1, pan‐keratin (AE1/AE3/PCK26), CK5/6, CK818, CK903, CD31, Factor VIII, CD15, D2‐40, EBER (in situ hybridization), CD163, and CD68.

The patient underwent bilateral explantation of her prostheses, followed by full‐body staging CT scans. There was a right supraclavicular lymphadenopathy, with three nodes measuring up to 4.1 cm in diameter. The chest wall and axilla showed extensive inflammation with subcutaneous gas. The tumor mass was associated with a right pleural effusion, which was found to contain only benign inflammatory cells and reactive mesothelial cells by pathologic examination. A bone marrow biopsy was negative for ALCL, but incidentally positive for a kappa clonal plasma cell population comprising 20% of the cellularity.

**Figure 1:**
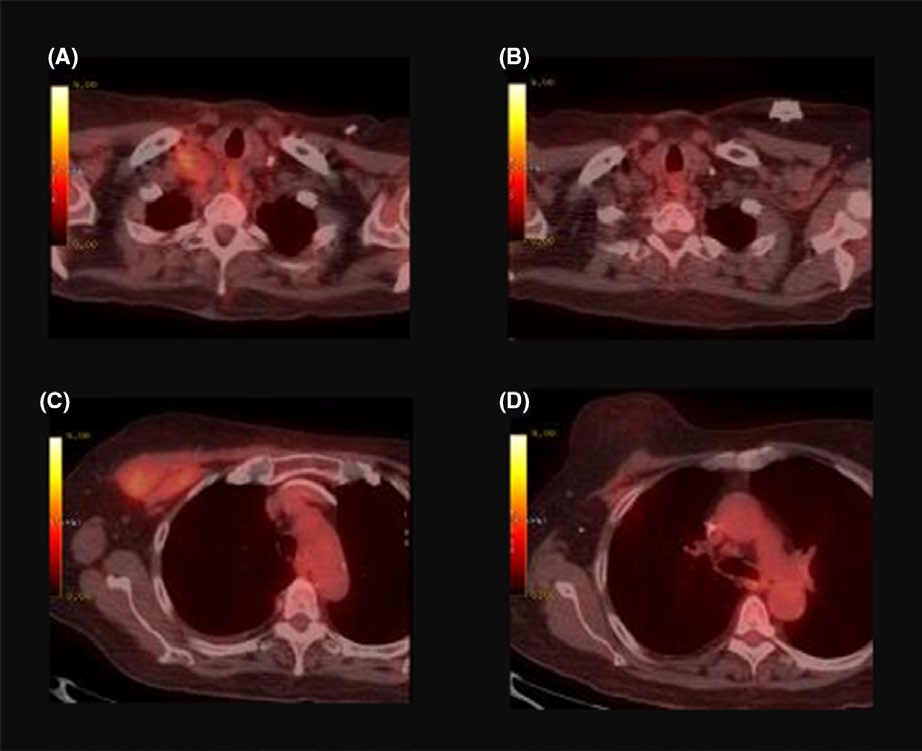
PET/CT images demonstrating the treatment response to brentuximab vedotin. A, Pretreatment hypermetabolic supraclavicular adenopathy. B, Resolution of supraclavicular adenopathy after the 5th cycle of BV. C, Hypermetabolic mass in the right anterior chest wall. D, Resolution of the mass after the 5th cycle of BV with formation of a large seroma (not shown)

The patient underwent palliative radiation therapy for persistent breast pain. Her case was presented to a multidisciplinary tumor board, and she subsequently began monotherapy with brentuximab vedotin (BV). Her dosing regimen was initiated at 1.8 mg per kilogram of body weight for 18 cycles at three‐week intervals. Her treatment was interrupted after her first cycle of BV due to a 12‐day hospital admission for multilobular pneumonia, colitis, and associated septic shock. Upon recovery, she resumed BV treatment and a subsequent PET/CT scan after her 5th cycle showed that the chest wall mass and supraclavicular adenopathy had resolved and a large seroma had formed in the anterior chest wall (Figure 1). She developed a peripheral neuropathy in her hands and feet after the 9th cycle, which was successfully treated with duloxetine and no dose reduction was required. At the writing of this case report, the patient is 20 months postcompletion of her 18‐cycle regimen and subsequent PET/CT scans continue to demonstrate complete remission.

## 3 DISCUSSION

While the exact incidence of BI‐ALCL is not known, it has been estimated between one and three cases per million women with breast implants.^8^ Age of onset ranges widely, with a mean in the 5th decade.^9^ The clinical presentation of BI‐ALCL is most frequently as a malignant effusion localized between a breast implant and the surrounding fibrous capsule. This is often associated with late‐onset seroma formation, breast asymmetry, and discomfort.^5^ While laboratory testing is typically not useful in the detection of BI‐ALCL, one case reported marked eosinophilia as the initial presentation^10^ and several markers of disease have been suggested for use in diagnostic testing of seroma fluid, such as JunB, SATB1, and FoxP3; all of which have been shown to be upregulated in BI‐ALCL.^11^

Unlike systemic ALCL, BI‐ALCL tends to follow an indolent course, with an overall 5‐year survival rate of 89%‐92%, compared to 30%‐49% and 70%‐86% in ALK‐ and ALK+ systemic ALCL, respectively.^6^, ^9^, ^12^ Patients presenting with a mass, indicating possible tumor extension beyond the implant capsule, typically have a worse prognosis. While studies differ in their estimates of survival for patients presenting with invasive disease, a cohort of 60 patients showed a 5‐year overall survival of 75% for patients presenting with a mass, compared to 100% for patients without a mass.^9^

There is no standard management protocol for BI‐ALCL. Several approaches to therapy have been suggested in the literature, including chemotherapy (most often based on the CHOP regimen, *cyclophosphamide, doxorubicin, vincristine, and prednisone*), radiation, and surgical excision. Of these options, it is clear that complete surgical excision of the breast implant, capsule, and any associated tumor mass provides the greatest benefit in terms of both morbidity and mortality.^6^, ^13^ The optimal management of unresectable, locally invasive disease remains unclear. Recent NCCN guidelines suggested brentuximab vedotin as an adjunctive therapy for invasive BI‐ALCL; however to date, its use has only been reported for low‐stage disease.^14^, ^15^

Brentuximab vedotin is a unique antibody‐drug conjugate, which received accelerated approval by the FDA in 2011 for the treatment of relapsed or refractory classical Hodgkin lymphoma (HL) and systemic ALCL. Since its introduction, the approved indications for BV have expanded to include previously untreated stage III/IV classical HL, consolidation therapy after autologous hematopoietic stem cell transplantation for classical HL and relapsed primary cutaneous ALCL or CD‐30 expressing mycosis fungoides.^16^ BV consists of a chimeric anti‐CD30 monoclonal antibody (SGN‐30) conjugated to the cytotoxic agent, monomethyl auristatin E (MMAE).^17^ CD30 is a type I transmembrane receptor protein of the TNF receptor superfamily, whose expression in benign tissues is limited to activated and virally infected lymphocytes and certain cells of the thymic medulla.^18^ It is expressed on a variety of cancerous cells, particularly Hodgkin and Reed‐Sternberg cells of Hodgkin lymphoma and diffusely in all types of ALCL; however, many other solid and hematologic malignancies have been found to express CD30 at various levels.^18^, ^19^ The function of CD30 in normal cells is poorly understood, as no human disease has been associated with defects in either CD30 or its ligand, CD153.^18^ The relative preponderance of this antigen on neoplastic cells and rarity of expression in healthy cells make CD30 an ideal target for immunotherapy. Upon binding of BV to CD30, the receptor‐antibody complex undergoes clathrin‐mediated endocytosis and lysosomal fusion.^20^ Within the lysosome, MMAE is released by proteolytic cleavage and acts to inhibit the assembly and polymerization of microtubules, causing G2/M cell cycle arrest and subsequent apoptosis.^18^ Some MMAE may then diffuse into the tumor microenvironment to further act on neighboring cells.

The most common adverse effect associated with BV is a peripheral sensory neuropathy, which can be dose limiting.^21^ Other less common and generally mild adverse effects include nausea, fatigue, rash, diarrhea, and cytopenias. Several serious, grade 3 to 4 reactions, including severe neuropathy, abdominal pain, pulmonary embolism, pneumonitis, and cytopenias may rarely occur.^16^ Our patient interrupted her treatment when she was hospitalized for 12 days with septic shock; however, given that she had only received one dose of BV prior to this, it is unlikely to have contributed. It should be noted that, compared with the substantial toxicity associated with CHOP and other cytotoxic regimens, the side effect profile exhibited by our patient was mild and well tolerated.

## 4 CONCLUSION

BI‐ALCL is a rare complication of breast implantation. Recent NCCN guidelines have been published, which suggest BV as a frontline option for extended disease; however, data reporting its efficacy for this indication is lacking. Here, we presented a case of unresectable, locally invasive BI‐ALCL in a 73‐year‐old woman, which was successfully treated with radiation, surgical explantation, and monotherapy with brentuximab vedotin. While larger studies are needed, the favorable tolerability profile and excellent treatment response demonstrated by our patient suggest that BV is a viable alternative to cytotoxic chemotherapy for invasive BI‐ALCL.

## CONFLICT OF INTEREST

The authors report no declarations of interest.

## AUTHOR'S CONTRIBUTION

AS: wrote the manuscript and prepared the images. IL: treated the patient and edited the text. Both authors approved the final manuscript.
